# Protein arginine methyltransferase 5 promotes bladder cancer growth through inhibiting NF-kB dependent apoptosis

**DOI:** 10.17179/excli2018-1719

**Published:** 2018-11-20

**Authors:** Guodong Hu, Xiu Wang, Yi Han, Ping Wang

**Affiliations:** 1 Department of Urology, the Affiliated Fourth Hospital of China Medical University, Shenyang, Liaoning, China; 2 Department of Urology, Shenyang Red Cross Hospital, Shenyang, Liaoning, China; 3 Department of Anesthesia, the Affiliated Fourth Hospital of China Medical University, Shenyang, Liaoning, China

**Keywords:** PRMT5, bladder cancer, NF-kB, apoptosis

## Abstract

Protein arginine methyltransferase 5 (PRMT5) has emerged as a key regulator of tumorigenesis. However, how PRMT5 functions in bladder cancer, the most common malignancy of the urological system, is unknown. We described here that PRMT5 is highly expressed in bladder cancer cell lines and primary human bladder cancer tissues. PRMT5 enhances the proliferation and colony formation of bladder cancer cells. PRMT5 knockdown induces bladder cancer cell apoptosis. Mechanistically, PRMT5 enhances NF-kB activation by targeting crucial anti-apoptotic genes such as *BCLXL* and *c-IAP1*, thereby inhibiting tumor cell apoptosis and sustaining proliferation. Importantly, PRMT5 inhibitor opposed tumor growth and *BCLXL* and *c-IAP1 *transcription in the bladder cancer xenograft model. Collectively, the current suggests the crucial role of PRMT5 as a promising therapeutic target in bladder cancers.

## Introduction

Bladder cancer is the most common urological carcinoma with about 430 000 cases and 165 000 deaths worldwide each year (Antoni et al., 2017[1]). Transitional cell carcinoma is the most common histological type of bladder cancer, accounting for more than 90 % of all cases (Torre et al., 2015[19]) . Radical cystectomy combined with chemotherapy is the major therapeutic strategy for bladder cancer patients, but the overall 10-year survival after radical cystectomy remains grave (Shen et al., 2009[17]; Witjes et al., 2014[22]). Therefore, the mechanism behind the progression and tumorigenesis of bladder cancer urgently requires investigation for development of novel therapeutic agents. 

RMT5 is a member of the protein arginine methyltransferase (PRMT) family and catalyzes arginine methylation by transferring methyl groups to arginine residues (Bedford and Clarke, 2009[2]; Blanc and Richard, 2017[3]). The modifications catalyzed by PRMT5 take an important role in lots of cellular processes (Karkhanis et al., 2011[13]; Yang and Bedford, 2013[24]). Extensive studies have shown that PRMT5 is overexpressed in varieties of human lymphoid malignancies and solid tumors, and it facilitates survival and proliferation of cancer cells (Chan-Penebre et al., 2015[5]; Harris et al., 2014[8]; Karkhanis et al., 2011[13]). PRMT5 induces arginine methylation of tumor suppressor p53 on the arginine 333,335 and 337 and affects the p53 target gene specificity, thereby escaping p53 surveillance and leading to tumorigenesis (Jansson et al., 2008[10]; Li et al., 2015[14]). PRMT5 potently activates NF-кB by dimethylation of its p65 subunit on arginine 30 (Wei et al., 2013[21]). It's well recognized that PRMT5 contributes to malignant processing, however, its role in bladder cancer is never reported.

In the present study, we showed for the first time a potential role of PRMT5 in promoting bladder cancer cell proliferation and tumor growth in *vitro *and in *vivo*. Mechanistically, PRMT5 promotes NF-κB recruitment on the promoter of anti-apoptotic targets *BCLXL *and *c-IAP1*. Our work thus demonstrated PRMT5 is crucial for facilitating bladder cancer growth, and is an important target for cancer therapy. 

## Materials and Methods

### Cell culture, plasmids and lentivirus

T24 and UM-UC-3 were from ATCC and were maintained in RPMI 1640 (Invitrogen) with 10 % fetal bovine serum (Invitrogen), 100 U/ml penicillin, and 100 U/ml streptomycin, in 5 % CO_2_ at 37 °C. The primary human bladder cancer tissues were obtained from patients with invasive transitional carcinoma. 293T cells were transient transfected with human PRMT5 shRNA (Dharmacon) with Lipofectamine and plus reagent (Invitrogen) to produce lentiviral supernatant. T24 and UM-UC-3 cells were infected with shPRMT5 lentivirus to stably overexpress PRMT5 shRNA.

### Western blot analysis

Western blot was carried out as previously described (Li et al., 2010[15]). Antibodies used were: PRMT5 (PRMT5-21, Santa Cruz), cleaved caspase-3 (5A1E, CST) and GAPDH (14C10, Cell Signaling Technology).

### Cell proliferation and colony formation assay

1 × 10^6^ cells were seeded in 10 cm dishes in RPMI 1640 containing 10 % FBS. Cell numbers were counted in 2 and 4 days. For colony formation assay, 50/well cells were placed in six-well plates in RPMI 1640 containing 10 % FBS for 10 days. Colonies were fixed with methanol and stained with 0.1 % crystal violet in 20 % methanol for 15 min.

### Annexin V apoptosis assay 

The cells were stained with PE-annexin V/7-AAD according to the manufacturer's instruction (BD Biosciences).

### Small interfering RNA (siRNA) 

siGENOME SMART pool targeting PRMT5 or control random siRNA were from Dharmacon. T24 cells were transfected with 50 nM siPRMT5 by Lipofectamine RNAi MAX reagent (Invitrogen).

### Luciferase assay 

p5XIP10 [21] was transfected in T24 cells using Lipofectamine and Plus Reagents (Invitrogen). 48 hours later, the NF-кB luciferase activity was monitored (Reporter Lysis Buffer kit, Promega).

### Quantitative PCR 

Total RNA was extracted using the RN-easy FFPE Kit (Qiagen) or RNeasy Mini kit (Qiagen). cDNA was prepared by the SuperScript III First-Strand Synthesis Kit (Invitrogen). qPCR was performed using SYBR Green Master Mix (Appliedbiosystems). Primers were designed by Primer3 (v.0.4.0) 

### Chromatin immunoprecipitation (ChIP) 

The truChIP Chromatin Shearing Kit (Covaris) was used to prepare chromatin. Chromatin was sheared into 200- to 700-bp fragments using a Covaris S2 instrument (duty cycle, 2 %; intensity, 3; 200 cycles per burst; 4 min). The IgG and NF-kB p65 antibodies (2A12A7, Thermo Fisher) were used for immunoprecipitation by the Quick Chip Kit (Imgenex SYBR Green Master Mix, (Appliedbiosystems) was used for qPCR to quantify precipitated DNA). The primers are from Qiagen EpiTect ChIP qPCR primers.

### Xenograft study

1 × 10^6^ T24 cells were injected subcutaneously into C57BL/6J mice (Shanghai Laboratory Animal Center) in the right flank. Tumor volume was measured using calipers and calculated as follows: volume = longest tumor diameter × (shortest tumor diameter)^2^/2. EPZ015666 or control vehicle (PBS) was administered orally twice daily (50 mg/kg) from 14 days after injection. Mice were sacrificed 28 days after injection. 

### Statistical analysis

Data were analyzed using GraphPad Prism 7. The statistical difference was calculated by using two-tailed Student *t* test or one-way ANOVA. A *P*-value below 0.05 was considered significant.

## Results

### PRMT5 is overexpressed in bladder cancer

**Figure 1:**
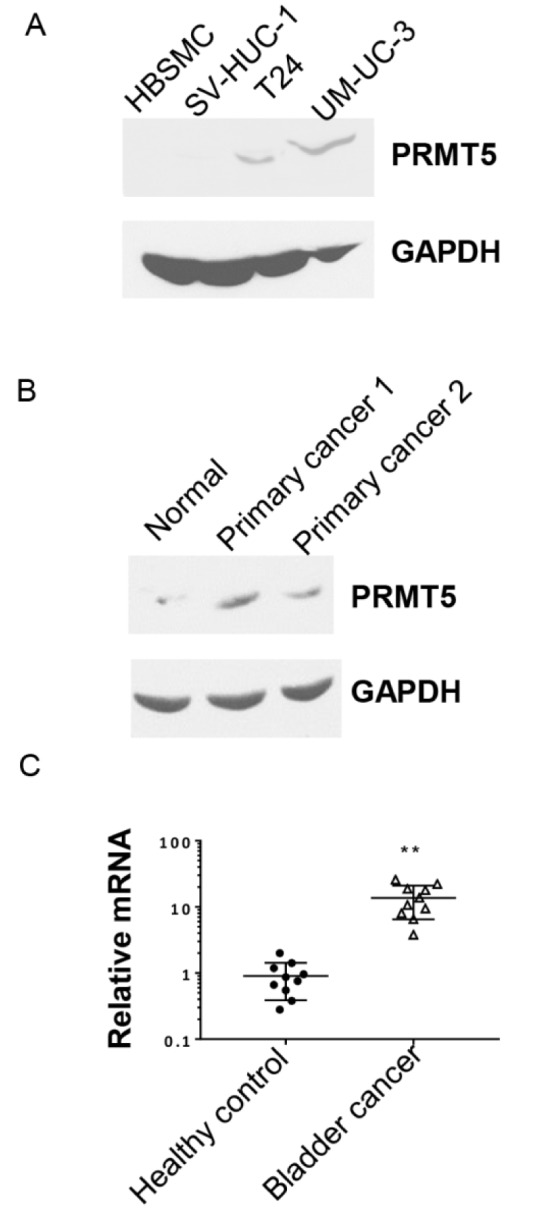
Expression level of PRMT5 is higher in bladder cancer cell lines and primary tissues. A. Western blot of PRMT5 protein in bladder cancer cells (T24 and UM-UC-3) and control normal bladder cell lines (HBSMC and SV-HUC-1). B. PRMT5 expression level in primary bladder cancer tissues (primary cancer 1 and primary cancer 2) and control normal bladder cancer adjacent tissue (normal). C. The relative expression of *PRMT5* in 10 pairs of FFPE samples from bladder cancer patients and healthy controls was analyzed by qRT-PCR. ** P < 0.01* vs. *healthy control.

To examine if PRMT5 is a tumor promoter in bladder cancer, we first tested PRMT5 protein expression in bladder cancer cell lines. As illustrated in Figure 1A(Fig. 1), compared to human normal bladder cell lines HBSMC and SV-HUC-1, PRMT5 protein levels were much higher in human bladder cancer cells (T24 and UM-UC-3). The primary human bladder cancer tissues were further used to detect PRMT5 protein levels. We showed that PRMT5 levels were notably higher in both primary bladder cancers, as compared to the normal adjacent tissue (Figure 1B(Fig. 1)). Furthermore, *PRMT5* transcription was also up-regulated in bladder cancer FFPE samples, as compared to samples from healthy control (Figure 1C(Fig. 1)). These data indicated that PRMT5 is substantially overexpressed in bladder cancer.

### PRMT5 facilitates bladder cancer cell growth

**Figure 2:**
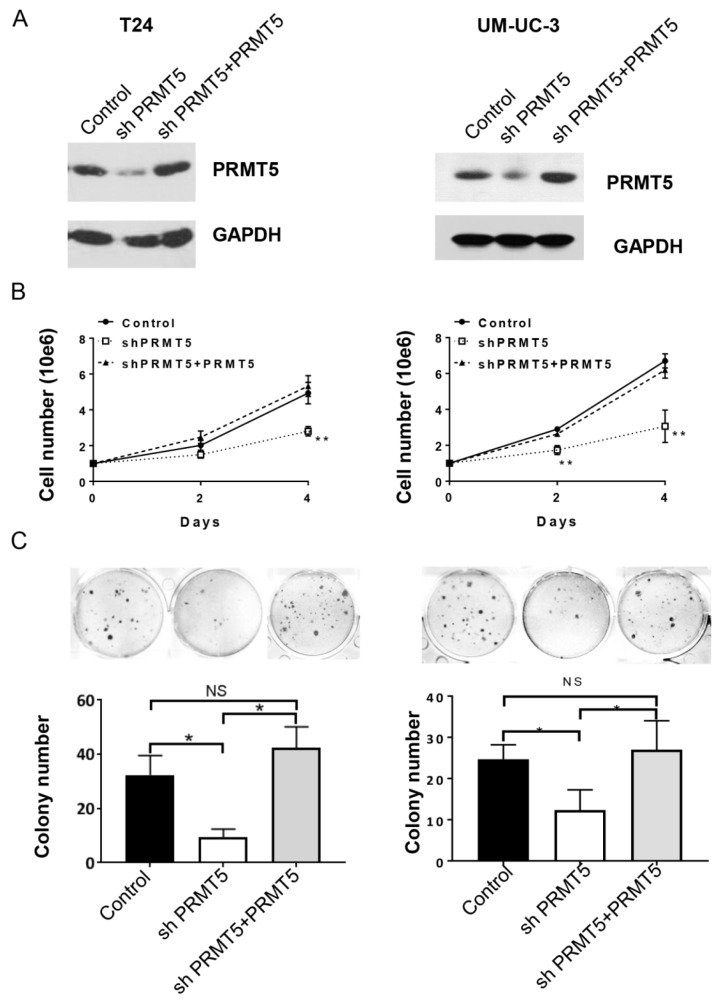
PRMT5 promotes bladder cancer cell proliferation. A. shPRMT5 knockdown and overexpression of PRMT5 in T24 (left) and UM-UC-3 (right) cells. B. Cell numbers in the shPRMT5 knockdown and overexpression of PRMT5 cells. C. The influence of PRMT5 knockdown on the cell survival of T24 (left) and UM-UC-3 (right) cells are measured by the clonogenic survival assay. * P < 0.05, ** P < 0.01*.*

PRMT5 is highly expressed in bladder cancer. Next, we knockdown the endogenous PRMT5 protein to investigate its role on bladder cancer growth. We detected the effect of PRMT5 knockdown on cell proliferation and colony formation of T24 and UM-UC-3 cells. shPRMT5 lentivirus were transduced into T24 and UM-UC-3 cells to establish stable cells overexpressing PRMT5 shRNA. Western blot confirmed the striking knockdown efficiency of shPRMT5 (Figure 2A(Fig. 2)). We further demonstrated that knockdown of PRMT5 in T24 and UM-UC-3 cells with shRNA resulted in much slower growth curve (Figure 2B(Fig. 2)), strongly suggesting that PRMT5 is the promoter for cell proliferation. Furthermore, shPRMT5 knockdown led to a dramatic decrease in the colony number in T24 and UM-UC-3 cells (Figure 2C(Fig. 2)), supporting the oncogenicity of PRMT5. Re-expression of PRMT5 in the shPRMT5 cells re-increased the potential of proliferation and colony formation in bladder cancer cells, further confirming the crucial role of PRMT5 in the neoplastic growth of bladder cancer (Figure 2A, 2B and 2C(Fig. 2)). These data collectively provide strong evidence for the tumor promoting potential of PRMT5 in human bladder cancer.

### PRMT5 knockdown leads to apoptosis of bladder cancer cells

**Figure 3:**
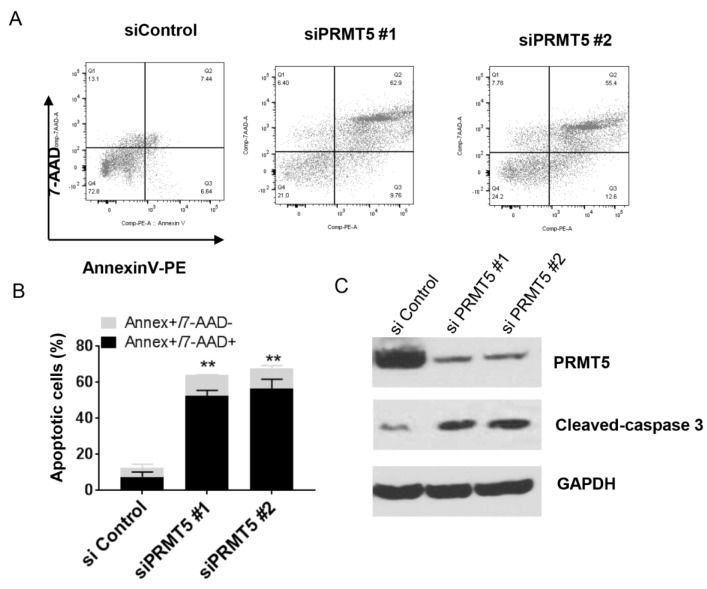
PRMT5 knockdown increases apoptosis in bladder cancer cells. T24 cells were transfected with siPRMT5 (#1or #2) or control siRNA (siControl) for 48 h. A. Annexin V/7-AAD staining of T24 cells. B. Statistics of Figure A. ** P < 0.01* vs. *control. C. Western blots for PRMT5 and caspase-3 protein expression.

We next detected if apoptosis contributes to decreased cell proliferation in PRMT5 knockdown bladder cancer cells. Flow cytometry showed the proportions of apoptotic cells by Annexin V/7-AAD staining (Figure 3A(Fig. 3)). 48 h after siPRMT5 transaction, the percentage of apoptotic cells was substantially enhanced compared with the sicontrol treated cells (Figure 3B(Fig. 3)). We further proved the cell apoptosis by Western blot, which showed increased protein levels of cleaved-caspase-3 in response to PRMT5 siRNA (#1 and #2). These results demonstrated that PRMT5 knockdown resulted in apoptosis in bladder cancer cells.

### PRMT5 inbibition suppresses NF-κB activativity and its target gene expression

**Figure 4:**
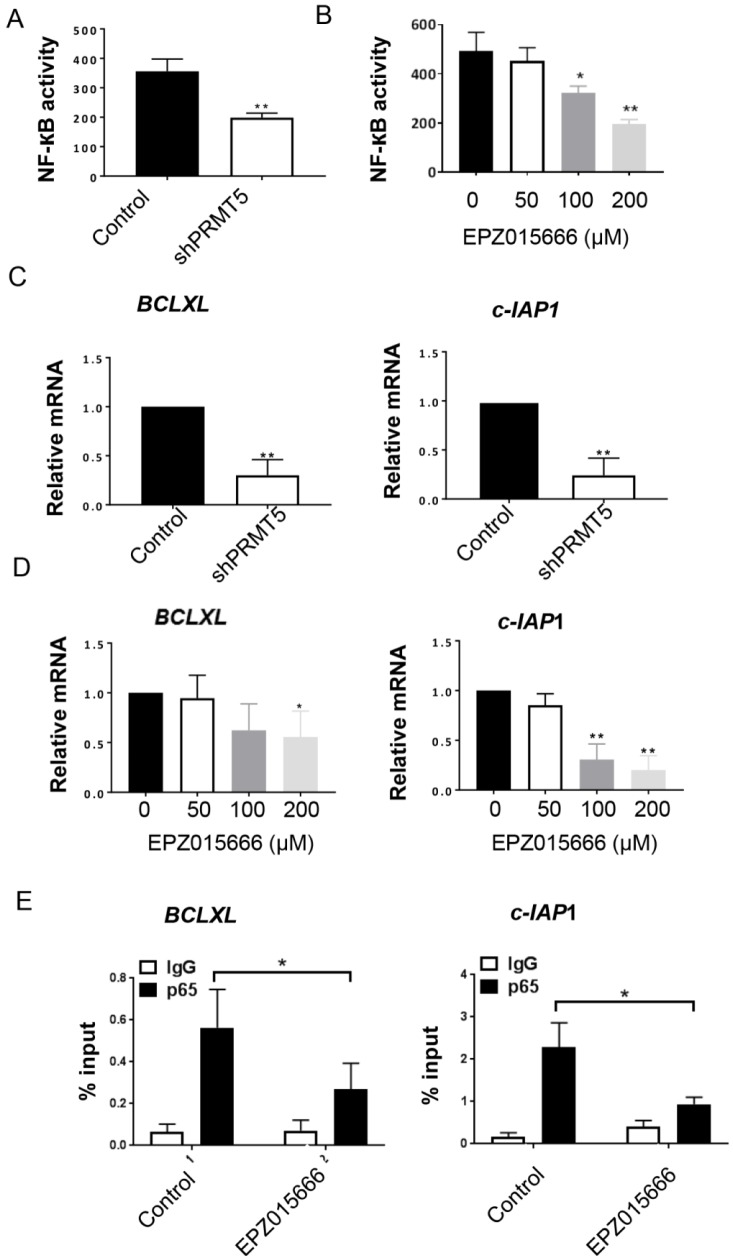
PRMT5 inhibition induces NF-кB inactivation in bladder cancer. A. shPRMT5 reduced NF-κB activation in T24 cells. B. EPZ015666 decreased NF-κB activation in a dose-dependent manner. C. shPRMT5 reduced NF-κB target genes (*BCLXL *and *c-IAP1*) expression. D. EPZ015666 decreased *BCLXL *and *c-IAP1 *expression. *P < 0.05 *vs. *control group. E. EPZ015666 (200 µM) impairs NF-κB enrichment on the *BCLXL* and *c-IAP1* promoters. ChIP was performed using IgG or NF-κB p65 antibody; *, *P* < 0.05; **, *P* < 0.01* vs. *control.

PRMT5 induces NF-κB activation by dimethylating R30 of the p65 subunit (Wei et al., 2013[21]). In Figure 4A(Fig. 4), we showed that PRMT5 inhibition by shRNA knockdown led to significant NF-κB inactivation. We used PRMT5 inhibitor, EPZ015666, to further determine its role on NF-κB activity. As indicated in Figure 4B(Fig. 4), 100 and 200 µM of EPZ015666 caused supression in NF-κB activity in T24 cells. The NF-κB transcription family is crucial for multiple gene transcription in cell growth, apoptosis and neoplastic transformation. To identify the relevant target genes that are controlled by the PRMT5-NF-κB activation axis, we detected some of the NF-κB candidate genes by qRT-PCR. Our data suggested that *c-IAP1* and *BCLXL *were strikingly suppressed in the shPRM5 knockdown T24 cells (Figure 4C & 4D(Fig. 4), left panels). Similarly, PRMT5 inhibitor EPZ015666 significantly inhibited NF-κB activation and its target gene trasncription in T24 bladder cancer cells (Figure 4C & 4D(Fig. 4), right panels). Overall, these results support a model in which NF-kB-dependent anti-apoptotic genes *c-IAP1* and *BCLXL* are crucial for PRMT5-mediated tumor growth in human bladder cancer.

Chromatin immunoprecipitation (ChIP) was performed using T24 bladder cancer cells treated with EPZ015666 to address critical roles of PRMT5 on NF-кB recruitment. Pharmacological inhibition of PRMT5 by EPZ015666 significantly impairs NF-κB p65 occupancy on *c-IAP1* and *BCLXL *promoters, (Figure 4E(Fig. 4)), providing further molecular insights into PRMT5 function. 

### Pharmacological inhibition of PRMT5 regulates apoptosis and impedes tumor growth in vivo

To confirm the essential role of PRMT5 as a therapeutic target in vitro, we investigated the effect of EPZ015666, a selective PRMT5 inhibitor (Braun et al., 2017[4]; Chan-Penebre et al., 2015[5]), on bladder tumor growth.

**Figure 5:**
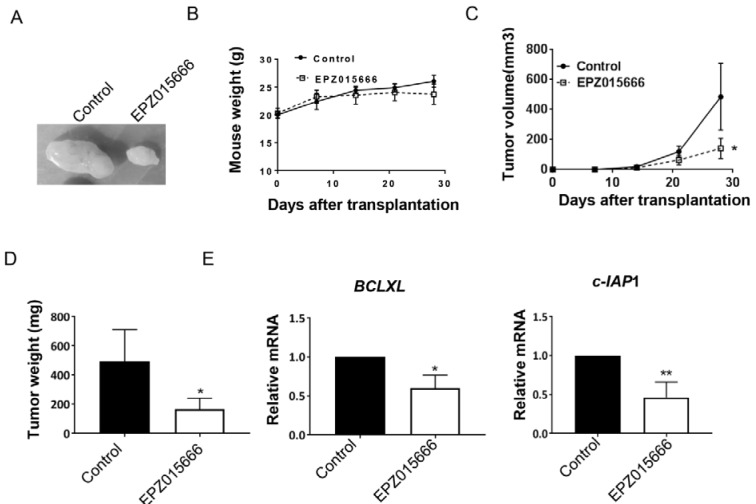
Pharmacological inhibition of PRMT5 has strong anti-tumor effects. T24 cells were injected into mice. EPZ015666 were administrated 14 days after injection. A. Morphology of subcutaneous tumors at 4 weeks after transplantation. B. Body weight of mice dosed with oral EPZ or vehicle (Control). C. Tumor volume over time as indicated. D. Tumor weight at 4 weeks after transplantation. E. qRT-PCR analysis of mRNA level of *BCLXL* and *c-IAP1* in excised tumors. *P < 0.05, ** *P* < 0.01 vs. control.

Administration of EPZ015666 did not lead to any body weight loss in tumor-bearing mice (Figure 5B(Fig. 5)). However, a 14-day EPZ015666 treatment resulted in significant reduction in tumor volume (Figures 5A and 5C(Fig. 5)). Moreover, EPZ015666 caused remarkable decrease of *BCLXL* and *c-IAP1* transcription (Figure 5D(Fig. 5)), confirming our *in vitro* findings concerning the associations between NF-кB activation, *BCLXL/c-IAP1* downregulation and apoptosis induced by genetic and pharmacological and genetic inhibition of PRMT5. We therefore propose that PRMT5 is a promising drug target in bladder cancer. 

## Discussion

Protein arginine methyltransferase 5 (PRMT5) is a member of the PRMT family of proteins, which symmetrically is dimethylating histones and non-histone protein. It's highlighted that PRMT5 plays central roles in the regulation of diverse cellular signaling and gene expression. PRMT5 protein levels are frequently high in numerous human cancers, such as lymphoma, glioblastoma, esophageal cancer, lung cancer, etc (Braun et al., 2017[4]; Chan-Penebre et al., 2015[5]; Jing et al., 2018[11]; Yan et al., 2014[23]). Overexpression of PRMT5 promotes proliferation, while inhibition of PRMT5 impedes cell growth (Karkhanis et al., 2011[13]; Yang and Bedford, 2013[24]). 

The recent report indicated that PRMT5 overexpressing causes tumor suppressor p53 function loss, further implying PRMT5's critical role as an oncoprotein (Li et al., 2015[14]). Nevertheless, the role of PRMT5 in bladder cancer, to our knowledge, is never reported. Notably, in the current study, overexpression of PRMT5 was observed in both human bladder cancer cell lines and patients. Our findings that PRMT5 inhibition induced cell apoptosis and growth suppression further confirmed that PRMT5 contributes directly to manifestation of the malignancy in human bladder cancer.

The NF-κB transcription family plays substantial roles in tumorigenesis as well as in the immune and inflammatory system. NF-κB signaling pathway consists of the most common heterotrimer of p50 and p65, which bind to its inhibitory protein IκBα. IκBα is the inactive form, masking the nuclear localization signal (Karin, 2006[12]; Lu and Stark, 2015[16]). Posttranscriptional modification of IκBα such as phosphorylation, ubiquitination, and degradation results in the release of the p50-p65 subunits from IκB and their nuclear translocation, generally causing transcription activation of multiple target genes (Lu and Stark, 2015[16]). It's reported that PRMT5 binds to the TRAIL receptor and thus contributed to TRAIL-induced NF-κB activation by Tanaka et al. (2009[18]). Multiple groups have proved that symmetrical dimethylation of NF-κB p65 by PRMT5 reinforces NF-κB transactivation activity (Harris et al., 2014[8], 2016[9]; Wei et al., 2013[21]). We found here that PRMT5 can reduce bladder cancer cell apoptosis by upregulating NF-κB activity, thereby potentiating cellular proliferation. We further confirmed the tumorigenic function of the PRMT5/NF-κB axis *in vivo* using xenograft tumor models. Our results demonstrate the carcinogenic function of the PRMT5/NF-κB signaling in bladder cancer and the role of PRMT5 inhibitor as a potent agent for the strategic therapy for bladder cancer.

It's well established that NF-κB activation plays central roles in the regulation of apoptosis (Van Antwerp et al., 1996[20]). The anti-apoptotic target genes of NF-κB trigger the immune and genomic surveillance system to exclude the malignant and pre-malignant cells. These anti-apoptotic genes include Bcl-2, FLIP and XIAP (inhibitors of apoptosis) families (Fan et al., 2008[7]). NF-κB diminishes TNF-induced JNK activation and apoptosis by intensifying XIAP and GADD45β expression (De Smaele et al., 2001[6]). Our *in vitro* and *in vivo* results showed that PRMT5 inhibition is a major driver for activation of NF-kB-dependent anti-apoptotic genes *c-IAP1* and *BCLXL *by NF-kB enrichment on the target promoter, providing mechanistic insights into its contribution to tumorigenesis and its influential to determine its potent as a therapeutic target.

In conclusion, our work illustrated that PRMT5 is highly expressed in bladder cancer cell lines and patients. Importantly, PRMT5 inhibition significantly represses bladder cancer cell proliferation and invasive bladder cancer growth by activation of critical NF-кB dependent anti-apoptotic genes. These findings indicated that PRMT5 may be an essential biomarker or drug target of bladder cancer.
